# Supplementary material for: Unhealthy yet Avoidable—How Cognitive Bias Modification Alters Behavioral and Brain Responses to Food Cues in Individuals with Obesity
Source: Nutrients. 2019 Apr 18;11(4):874. doi: 10.3390/nu11040874 (PMC6521098; doi:10.3390/nu11040874)
Supplement: Supplementary file 1 [file nutrients-11-00874-s001.pdf]

nutrients

Supplementary results

Table 1. Questionnaire differences between the training and no-training groups; p-values reflect significance of group differences.

	Training Group	No-Training Group	p-value/t(31) value	Effect size |d|
	Mean/SD			
TFEQ cognitive control	8.11/6.12	6.63/4.01	0.417/-0.822	0.286
TFEQ disinhibition	9.29/3.16	7.19/3.56	0.082/-1.800	0.624
TFEQ hunger	6.94/4.35	5.75/3.44	0.391/-0.869	0.303
BIS	20.53/4.61	19.94/5.23	0.732/-0.345	0.120
BAS drive	12.00/2.06	11.62/2.63	0.651/-0.457	0.161
BAS fun seeking	13.18/2.16	12.38/1.89	0.267/-1.131	0.394
BAS reward responsivity	16.76/1.92	16.75/1.39	0.980/-0.025	0.006

Table S2. Ratings of healthy and unhealthy images used in the AAT paradigm for healthiness and liking on a scale from 0 to 10.

Image category	Scale	Training Group	Sham-training Group	p-value/t(31)-value	Effect size |d|
	Mean/SD				
Unhealthy	Liking	6.11/0.90	5.95/1.34	0.692/-0.399	0.140
	Healthiness	1.81/0.78	1.76/1.21	0.885/-0.146	0.049
Healthy	Liking	7.64/0.71	7.44/1.17	0.555/-0.596	0.207
	Healthiness	8.67/0.67	8.73/0.62	0.775/0.288	0.093

Table 3. Picture-sorting task – ratings of healthy and unhealthy images on healthiness and liking on a scale from 0 to 10. We observed no significant group differences.

Image Category	Scale	Training Group		Sham-training Group		Image category * time * group interaction	Effect size η^2_p
		Pre	Post	Pre	Post		
		Mean/SD				p-value/F(1,31)-value	
Liking	Unhealthy	5.76/ 1.17	5.37/ 1.57	5.97/ 1.82	5.39/ 1.94	0.975/0.001	0.014
	Healthy	7.24 /0.99	7.39/ 1.07	7.23/ 1.63	7.35/ 1.63		
Healthiness	Unhealthy	1.38/ 0.82	1.53/ 0.88	1.25/ 1.01	1.50/ 1.16	0.503/0.453	0
	Healthy	8.42/ 0.76	8.43 /0.83	8.27/ 0.97	8.38/ 0.90		
